# Optimized Intrusion Detection for IoMT Networks with Tree-Based Machine Learning and Filter-Based Feature Selection

**DOI:** 10.3390/s24175712

**Published:** 2024-09-02

**Authors:** Ghaida Balhareth, Mohammad Ilyas

**Affiliations:** Department of Electrical Engineering & Computer Science, Florida Atlantic University, 777 Glades Road, Boca Raton, FL 33431, USA; ilyas@fau.edu

**Keywords:** machine learning algorithms, feature selection, intrusion detection system (IDS), IoMT, SoT, XGBoost, CatBoost

## Abstract

The Internet of Medical Things (IoMTs) is a network of connected medical equipment such as pacemakers, prosthetics, and smartwatches. Utilizing the IoMT-based system, a huge amount of data is generated, offering experts a valuable resource for tasks such as prediction, real-time monitoring, and diagnosis. To do so, the patient’s health data must be transferred to database storage for processing because of the limitations of the storage and computation capabilities of IoMT devices. Consequently, concerns regarding security and privacy can arise due to the limited control over the transmitted information and reliance on wireless transmission, which leaves the network vulnerable to several kinds of attacks. Motivated by this, in this study, we aim to build and improve an efficient intrusion detection system (IDS) for IoMT networks. The proposed IDS leverages tree-based machine learning classifiers combined with filter-based feature selection techniques to enhance detection accuracy and efficiency. The proposed model is used for monitoring and identifying unauthorized or malicious activities within medical devices and networks. To optimize performance and minimize computation costs, we utilize Mutual Information (MI) and XGBoost as filter-based feature selection methods. Then, to reduce the number of the chosen features selected, we apply a mathematical set (intersection) to extract the common features. The proposed method can detect intruders while data are being transferred, allowing for the accurate and efficient analysis of healthcare data at the network’s edge. The system’s performance is assessed using the CICIDS2017 dataset. We evaluate the proposed model in terms of accuracy, F1 score, recall, precision, true positive rate, and false positive rate. The proposed model achieves 98.79% accuracy and a low false alarm rate 0.007 FAR on the CICIDS2017 dataset according to the experimental results. While this study focuses on binary classification for intrusion detection, we are planning to build a multi-classification approach for future work which will be able to not only detect the attacks but also categorize them. Additionally, we will consider using our proposed feature selection technique for different ML classifiers and evaluate the model’s performance empirically in real-world IoMT scenarios.

## 1. Introduction

The rapid expansion of the Internet has given rise to the Internet of Things (IoT), a paradigm used to create smart environments involving several technological fields and their associated applications, such as smart cities and homes [1,2]. Within this context, incorporating healthcare sensors and devices into the IoT has accelerated the development of the Internet of Medical Things (IoMT) [3]. The IoMT enables remote monitoring in healthcare, enhancing patient safety and facilitating more effective interactions between patients and physicians, inspiring clinicians to provide exceptional treatment [4,5]. Various sensors in IoMT systems collect real-time, sensitive patient data to provide medical practitioners with a better understanding of critical situations and empower patients with knowledge about their health conditions [5].

The healthcare industry is experiencing a digital revolution that has the potential to revolutionize patient care and health data [6,7]. The challenges posed by the COVID-19 pandemic, which necessitated the use of digital technology to support the mental well-being of healthcare professionals remotely [8,9], accelerated this revolution and caused an increase in attacks on the healthcare industry [6].

Biosensors, including motion, breathing, temperature, vision, and blood pressure sensors, play a crucial role in connecting individuals to healthcare systems [10,11]. These sensors have the capability to be implanted into the human body and produce massive amounts of data in real-time. The IoMT landscape is witnessing exponential growth [7,12]. In 2022 [13], the global Internet of Medical Things (IoMT) market was valued at $48.7 billion and is expected to grow significantly, reaching $370.9 billion by 2032.

However, this surge in demand necessitates advancements in data storage, processing, and security measures. To handle the vast volume of data and address security concerns, artificial intelligence technologies such as machine learning (ML) are employed for decision analysis, classification, and the detection of cyber-attacks [14]. ML is the optimal method for ensuring the security and stability of IoT network traffic [15]. Furthermore, it is a promising approach for handling security vulnerabilities and hidden attacks in healthcare systems. ML techniques can identify attacks by monitoring data modifications or detecting changes in network traffic features [7]. Despite the benefits, the rapid expansion of IoMT systems has exposed critical vulnerabilities, making many devices susceptible to cyber-attacks that pose potential risks to patients’ lives [16]. Furthermore, by 2025, the estimated cost of cybercrimes will increase to USD 10.5 trillion [17,18]. The open wireless communication mediums used by IoMT devices coupled with a virtual and connected healthcare environment transmitting potentially unsecured data create a breeding ground for cyber-attacks [16].

Weak authentication measures and poor design contribute to unauthorized access, allowing attackers to remotely manipulate drug doses or turn IoMT sensors into botnets for denial-of-service attacks [19]. Incidents like the 2018 ransomware cyber-attack on an Indiana hospital system highlight the significant financial and operational impact of such security breaches [2,20]. In another incident in 2023, Prospect Medical Holdings had a ransomware attack that shut down and disrupted emergency services in multiple U.S. states, requiring FBI action. The incident shows how the healthcare industry is susceptible to cybercrime. Consequently, the attack resulted in substantial interruptions to critical services, including elective surgeries, outpatient visits, blood drives, and ambulance operations [21]. In a cyber-attack in response to growing security challenges, IDSs have emerged as a crucial component of Security of Things (SoT) paradigms and have shown positive results in addressing the difficulties associated with the IoMT, such as the limited availability of resources, delays in data transmission, the ability to handle large-scale operations, and diversity [22,23]. IDSs help detect anomalies or intrusions in live network traffic, which is critical for protecting sensitive health data in IoMT systems [22,24]. As the complexity and incompatibility of IoMT sensors contribute to security issues, an efficient and robust security mechanism is imperative to mitigate cyber-attacks in the highly scalable and distributed IoMT environment [25].

To enhance the effectiveness of IDSs, various ML and feature selection methods have been implemented. These methods aim to achieve optimal detection rates and minimize false alarm rates. The objective of this research is to propose a model for building and improving an efficient IDS for IoMT networks. Our model utilizes tree-based machine learning classifiers combined with advanced feature selection methods to ensure high detection accuracy. The key contributions of this study include the following:

- We propose an optimized Intrusion Detection for IoMT Networks with tree-based machine learning on a balanced CICIDS2017 dataset. These algorithms include Decision Tree, Random Forest, XGBoost, and CatBoost.
- We apply filter-based feature selection methods to determine the relative importance of features. These methods are Mutual Information and the XGBoost method named (MI-XGBoost).
- We apply the mathematical set (intersection theory) concept to find the most common features between the chosen one after applying MI-XGBoost. This approach aims to extract the minimal set of the most relevant features.
- We evaluate the performance of our proposed model in terms of accuracy, F1 score, recall, precision, TPR, and FPR. Then, we use k = tenfold cross-validation to validate our model.
- We compare our model with recent related works.

The structure of this paper is organized as follows: Section 2 presents the related works. Section 3 presents our research methodology. Section 4 illustrates an overview of the proposed model. Section 5 shows the experimental tools employed in this study. The evaluation metrics and results are provided in Section 6. A comparison of the proposed model with other state-of-the-art methods is presented in Section 7. Finally, Section 8 concludes our paper.

## 2. Related Works

Numerous works have been carried out using machine learning (ML) to detect trends and behaviors in network traffic that could potentially indicate malicious behavior. Several state-of-the-art research studies have considered the security and privacy of IoT and IoMT systems in recent years. In this section, we review these papers that have attempted to improve IDS effectiveness in the face of growing network traffic and a wider variety of threats.

In [26], the authors suggest using a hybrid ensemble learning approach after a feature selection strategy. Their research focuses on applying Correlation Feature Selection and Forest Panelized Attributes (CFS–FPA) to reduce the dimensionality of the CICIDS2017 dataset. They aim to verify a hybrid ensemble scheme and identify the best ML approach among SVM, Random Forest, Naïve Bayes, and K-Nearest Neighbor (KNN) classifiers. By comparing classification strategies before and after AdaBoosting modification, the findings will improve the suggested feature selection strategy.

A novel minimized redundancy discriminative feature selection technique (MRD-FS) was introduced and applied by Albulayhi et al. [27] to address the problem of redundant features. The selection of discriminative features was based on two criteria: redundancy and representativeness. Their model was tested using the BoT-IoT dataset.

Using the CICIDS2017 dataset, the authors [28] studied the detection of multiple types of assaults, such as DDoS, Brute Force, DoS, Web Attack, Infiltration, Botnet, and Port Scan. In-depth dataset explanation, pre-processing, feature selection (RFE), and model validation using a variety of techniques were all part of their system. They stressed how important it is to use RFE in conjunction with data pre-processing for every assault while utilizing techniques such as Random Forest, SVM, SGD, GaussianNB, Decision Tree, KNN, and Logistic Regression. Notably, Random Forest outperformed other algorithms in binary and multi-class classification tasks, achieving the best accuracy. The study emphasizes how crucial feature selection methods and thorough pre-processing are to improving attack detection on a dataset.

An upgraded intrusion detection system (IDS) technique that combines SVM with Naïve Bayes (NB) feature embedding was presented by Gu et al. [29]. They transformed the original features using NB feature embedding to produce high-quality data for the training of SVM classifier. This method performed better than previous methods, with improved detection and false alarm rates and accuracy scores of 98.92% on CICIDS2017 and 99.35% on NSL-KDD datasets.

An IDS strategy utilizing several ML algorithms, including support vector machine (SVM), Decision Tree (DT), K-Nearest Neighbor (KNN), Naïve Bayes (NB), Logistic Regression (LR), and Random Forest (RF), was presented by Pankaj et al. [30]. RF performed better after feature selection using Pearson’s correlation coefficient, with an accuracy rate of up to 99.93%. This emphasizes how effective RF is at detecting intrusions in comparison to other supervised techniques.

The authors of [23] introduced and applied an innovative extraction method designed for IDSs. The method employs two entropy-based techniques to select significant features across multiple ratios: gain ratio (GR) and information gain (IG).

Kurniabudi et al. [31] presented an IG-based method that groups feature according to their weights after determining feature weights using IG. This approach, when paired with RF for classification, attained an impressive 99.86% accuracy. This demonstrates how IG works well for feature selection and how it works well with RF to provide correct classification.

In [32], security and privacy issues are addressed by an ML-based IDS designed for the Internet of Things (IoT). The suggested IDS was assessed using a variety of ML techniques, including CatBoost, KNN, SVM, NB, and XGBoost using the UNSW-NB15 dataset. Unexpectedly, XGBoost attained a high accuracy of 99.9%, highlighting how well it works to improve IoT security.

The text corpus’s redundant and irrelevant properties have a detrimental effect on text classification. Ref. [33] presented a hybrid filter-based feature selection method that combines IG with principal component analysis. In their investigation, they discovered that by selecting the appropriate feature subset and thereby cutting down on training time, their suggested feature selection strategy considerably reduces the data dimension.

Various solutions have been suggested for handling security and privacy concerns in smart medical networks, as these attacks harm patients’ healthcare and leave them at risk.

Within the Security of Things (SoT) paradigm, Iwendi et al. [22] presented an IDS that has applications in smart healthcare and influences medical infrastructures. They used Naive Bayes, RF, and Logistic Regression classifiers for ML on the NSL-KDD dataset. They improved their strategy by combining Random Forest with a weighted genetic algorithm, which allowed them to maximize detection rates while reducing false positives and true negatives. The integration produced a remarkable 98.81% accuracy rate.

A medical device and WBAN-based IDS were developed by Thamilarasu et al. [34] and tested extensively on subsets of the IoMT, such as WBANs. The system performed exceptionally well, with accuracy rates of 99.6% for network-level intrusion detection and 98.2% for device-level intrusion detection. The study simulated a hospital network topology.

An anomaly detection technique was established by Šabić et al. [35] with the express purpose of identifying anomalies in heart rate data. They assessed the data fit using five algorithms, including supervised and unsupervised techniques. In modeling such systems, their study showed that Random Forest and ensemble approaches were especially effective, achieving over 99% accuracy, indicating their potential for robust anomaly identification in heart rate data.

An advanced healthcare security system was developed in [24] to identify and mitigate spoofing threats and unauthorized data modifications. This system is comprised of an IDS, data gateway, server, and assaults. The researchers used a dataset that contained 14,000 non-attack and 2000 attack data samples to train the model. They employed four ML techniques: Random Forest, artificial neural network, KNN, and support vector machine (SVM). Their study showed that an artificial neural network demonstrates the highest C-statistic in recognizing traffic threats, ranging from 7% to 25%.

A research team suggested an IDS for anomaly detection in the Connected Healthcare System in [36] that is based on a stacked autoencoder (SAE). Their approach entails discretization and normalization of the data, and then an SAE is used to extract features. ML models are then supplied with the extracted characteristics to be classified. SVM, NB, KNN, and XGBoost were tested using actual patient data and simulated attacks (DoS, counterfeit, temper, and replay), with XGBoost demonstrating the greatest performance with 97.83% accuracy, 2.35% FPR, and 1.65% FNR.

A dataset called the Edith Cowan University-Internet of Health Things (ECU-IoHT) was developed and made available to the public by Ahmed et al. [16]. This dataset was subjected to cyber-attacks that targeted various vulnerabilities. The purpose of these attacks was to gather information about attack behaviors and assist in the creation of effective defense methods. According to their research, the KNN algorithm outperformed statistical clustering and kernel-based algorithms in terms of anomaly identification performance.

To identify cyber-attacks in IoMT networks, the author in [2] introduced an IDS based on ensemble learning within a fog–cloud architecture. Their solution entailed preparing traffic data by handling missing values, transforming categorical values to numerical ones, and applying the correlation coefficient method to choose characteristics. Next, predictions were produced by a learning set that includes NB, DT, and RF. These predictions were then combined by XGBoost using majority voting. Infrastructure as a Service was used in the cloud by the deployment architecture, and Software as a Service was used at the fog level. Evaluation was conducted using measures including accuracy, detection rate, precision, FAR, and F1-score on the Ton-IoT dataset, which represents heterogeneous and large-scale IoT networks. Outcomes exceeded previous IDS research, with a 99.98% detection rate, 96.35% accuracy, and a false alarm rate decrease of up to 5.59%. In the fog–cloud framework, an ensemble approach shows promise in improving cyber-attack detection in IoMT networks.

## 3. Research Methodology

The research methodology in this study is used to develop and evaluate the intrusion detection system for Internet of Medical Things networks. Our methodology focuses on using tree-based machine learning algorithms combined with an efficient feature selection method. The details of the proposed method are presented in the following section.

## 4. Overview of the Proposed Model

We propose optimized intrusion detection for IoMT networks with tree-based ML to detect and classify traffic into normal and abnormal traffic. The aim of this work is to enhance security and build an effective IDS by using supervised Tree ML algorithms. Four tree-based ML algorithms are applied in our model: Decision Tree (DT), Random Forest (RF), XGBoost, and CatBoost. Additionally, to reduce the computation cost and the number of features in our model, a filter-based feature selection technique was used. Finally, different evaluation metrics, namely, accuracy, recall, precision, F1-score, ROC, FPR, and TPR were used to evaluate the performance of the models. The dataset description and the flow of the proposed method are illustrated in detail below.

### 4.1. Dataset Description

**Figure 1:**
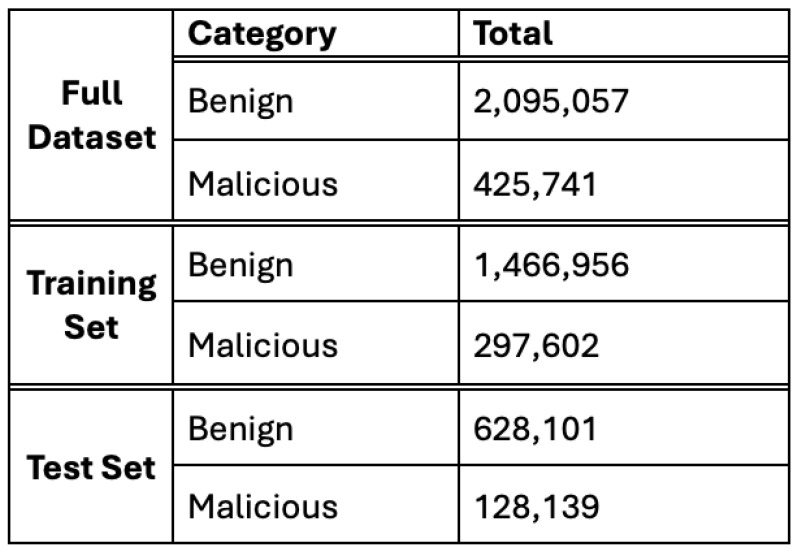
The distribution of the benign and malicious traffic of the CICIDS2017 dataset.

The Canadian Cybersecurity IDS Institute at the University of New Brunswick created the CICIDS2017 dataset utilized in this study. This dataset is commonly used and includes benign and the most recent prevalent cyber assaults, making it a significant resource for cybersecurity researchers [37]. This dataset is commonly utilized to create and evaluate intrusion detection systems and examine network security and anomaly detection. The CICIDS2017 dataset consists of eight separate files, each including both normal and malicious traffic data. The files document network activity for five days [37]. Table 1 represents the day activity and the traffic type for each file. Figure 1 depicts the distribution of the benign and malicious traffic of the used dataset after the pre-processing step.

### 4.2. The Flow of the Proposed Model

IoMT network faces significant security challenges, particularly in detecting intrusions that compromise sensitive data. In order to address this issue, we propose an optimized IDS using tree machine learning and filtering techniques. Our proposed method consists of three steps:

- Data cleaning and pre-processing: This is an important step for ensuring the accuracy and reliability of the proposed model. The CICIDS2017 dataset is often incomplete or imbalanced, which could affect the performance of the proposed IDS; thus, the dataset must be pre-processed. To do so, the initial step to pre-process the data in our model involves removing null values and removing duplicate records. Additionally, to ensure that all features contribute equally to the model’s training, we apply Max-Min to standardize the feature range. Finally, to deal with an imbalanced dataset, the class weight is used.
- Feature selection: Feature selection is a critical component of our proposed model. This step aims to reduce the computational complexity of the model and enhances its performance by focusing on the relevant features. Thus, we apply MI and XGBoost for feature selection. Then, we reduce the number of selected features by using a mathematical set theory intersection to create a unified subset of relevant features.
- Tree-based machine learning classification: We use well-known tree classifiers such as DT, RF, XGBoost, and CatBoost to classify network data into benign and malicious. These classifiers are chosen for our model based on their robustness in handling complicated and non-linear patterns in network traffic, as well as their efficiency in processing large amounts of data. By exploiting different aspects of the data, each classifier enhances the overall detection capability of the intrusion detection system (IDS) and increases the precision and dependability of our intrusion detection.

Then, we use the evaluation metrics to evaluate the proposed method’s performance. By focusing on the most relevant features and employing powerful classifiers, our model addresses the key security challenges in IoMT environments.

**Figure 2:**
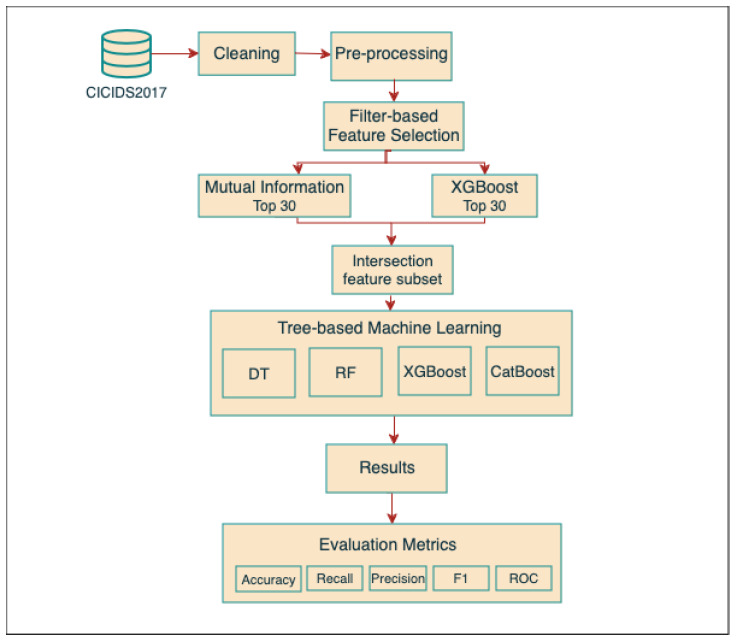
The flow of The proposed model.

Figure 2 illustrates the flow of the proposed model. The following describes each step in detail.

#### 4.2.1. Data Cleaning and Pre-Processing

**Figure 3:**
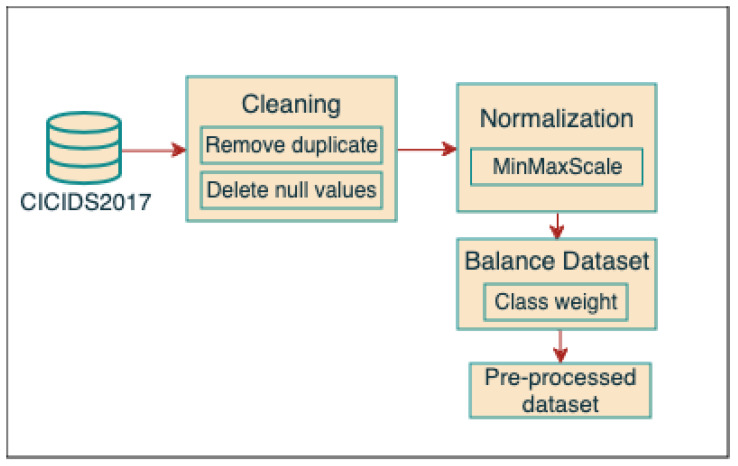
Illustration of the workflow of the data pre-processing steps.

Figure 3 demonstrates the workflow of the data pre-processing steps applied to the CICIDS2017 dataset.

1. Data Cleaning:
  After combining the eight files into a single dataset, various methods were employed on the CICIDS2017 dataset, including data cleansing and the elimination of null values and duplicates.
2. Data Pre-processing:
  As real-world data are often inadequate and mismatched, pre-processing the data is essential. Pre-processing the dataset is crucial for improving its quality by eliminating noise, addressing missing values, standardizing or normalizing features, and readying the data for analysis or model training. Many techniques have been proposed to pre-process data, including scaling. Scaling is a pre-processing technique that involves applying certain scalers to the numerical characteristics of a dataset to optimize it for machine learning algorithms [38]. The failure to utilize scaling for pre-processing the dataset may result in unsatisfactory model performance, skewed predictions, and erroneous interpretations of the model’s behavior. Scaling is a crucial pre-processing step in many machine learning workflows, as it guarantees dependable and precise model training and predictions. Thus, we first divided the dataset into 30% as a testing set and 70% as a training set, then Max-Min was used in the pre-processing step to scale the features of the dataset to a specified range, usually from 0 to 1 [39]. This technique standardizes the range of features to guarantee they are all on the same scale. This avoids larger-scale features from overpowering smaller-scale traits in model training. Additionally, to improve the performance of the model, class weight was used to address class imbalance issues in datasets where one class is significantly more common than the others. This involves assigning higher significance to data from the minority class and lower significance to samples from the majority class during model training. Eventually, Max-Min scaling standardizes the features, whereas class weight considers dataset imbalance. Collectively, they can improve the resilience and adaptability of the machine learning model. The main objective of using balanced data for model training is to increase the classification accuracy of the systems, thus boosting performance [40].

#### 4.2.2. Feature Selection

While each connection in a dataset is distinguished by several features, not all of these features are essential for building the IDS; hence, it is crucial to identify the most relevant features of traffic data. Feature selection techniques have several advantages, including but not limited to enhanced model performance, accelerated training and inference, cost reduction, etc. [41,42]. Thus, filter-based feature selection techniques, Mutual Information, and XGBoost are utilized in our model to choose the most significant and informative features from the dataset. Every filter method selects relevant features and ranks them based on their score. The subsections below illustrate the filter-based techniques used.

**Figure 4:**
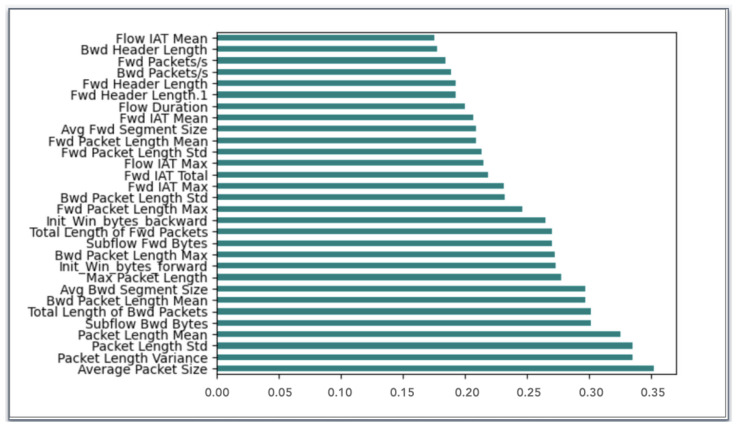
Features importance ranking by Mutual Information.

**Figure 5:**
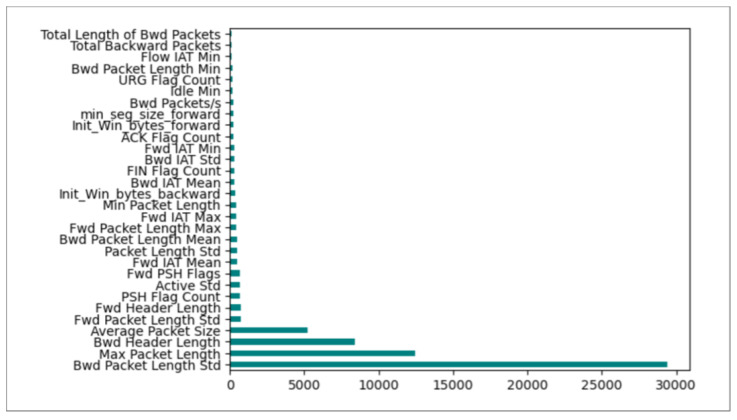
Features importance ranking By XGBoost.

- **Mutual Information**
  One of the most useful metrics for assessing the interdependence of variables is mutual information (MI). MI generates a non-negative result, and the two observed variables are statistically independent if the Mutual Information is zero. If it is greater than zero, it indicates a stronger relationship between the two variables [43]. Thus, we used it in our model to determine the most relevant features. Figure 4 presents the top 30 selected features by applying MI.
- **XGBoost**
  The fundamental concept of the XGBoost method is sequentially training a set of tree models, progressively improving their accuracy by including a penalty parameter to manage the model complexity [44]. The XGBoost technique computes a score for each feature of the used dataset. The measure is used to evaluate the importance of each feature. So, we can determine the importance of inputs in the learning and classification processes. Figure 5 demonstrates the top 30 features selected by applying XGBoost to the CICIDS2017 dataset.

The feature importance rankings obtained by MI and XGBoost, respectively, are shown in Figure 4 and Figure 5. The variations in these rankings are to be expected because each technique uses a different methodology to rank the features. By analyzing each feature’s statistical dependency with the target variable separately from other features, MI evaluates each feature’s importance. This method is able to identify features that have a direct, non-linear relationship with the target. Conversely, XGBoost assesses the significance of features by measuring their impact on reducing the error in a series of Decision Trees. In contrast to MI, this approach considers feature interactions, which may result in differential relevance rankings.

Utilizing the mathematical set theory (intersection) methodology, we address the differences between the two methods of the selected features and determine the most relevant features for our model. This method helps by concentrating on features that are consistently significant from various perspectives by identifying the point where the top features from the two methods intersected.

Thus, after employing our filter-based feature selection method, MI-XGBoost, on the CICIDS2017 dataset, the optimal attributes are then determined by employing mathematical set theory (intersection) from each base selector to create a unified feature subset. This approach aims to retain beneficial candidate features that are capable of boosting the classification accuracy. The intersection method efficiently integrates common features chosen by the two subsets generated from the base selectors into the resultant subset of acquired features. The common features that are present in both S1 and S2, where S1 and S2 represent the feature selections by MI and XGBoost, respectively. Thus, the feature subset intersection is expressed in Equation (1) as follows:

(1) $$f\in (S_{1}\cap S_{2})$$

where the variable ***f*** represents the features within the dataset. The overall intersection of the two subsets of features is defined in Equation (2):

(2) $$\cap \left\{S_{1},S_{2}\right\}=S_{1}\cap S_{2}$$

**Figure 6:**
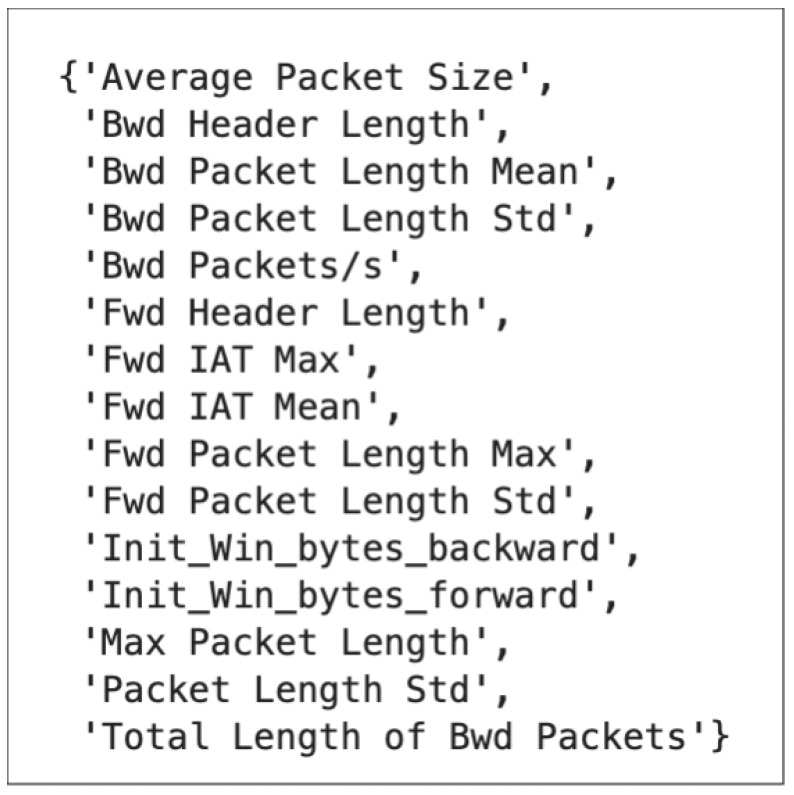
The common features between MI-XGBoost.

Using Equation (2), 15 features were selected as common features, which are used in our tree-based model as depicted in Figure 6.

Equation (1) represents the intersection of the top features identified by MI and XGBoost. The motivation behind this decision is to combine the strengths of both feature selection methods. Using the intersection technique, we focus on the features that are consistently important across both methods. We aim to create a reliable feature set that enhances the performance of our proposed IDS.

Considering the most relevant features from both methods, we find that the intersection provides a balanced and effective feature set that supports the overall goal of accurate and efficient intrusion detection.

#### 4.2.3. Tree-Based ML Classifiers

Using the CICIDS2017 dataset, our four tree-based ML classifier models are used to detect abnormal traffic in the network. We used the optimum feature set gained from the MI-XGBoost filter-based feature selection approach as inputs for our model, as shown in Figure 2. The output aims to detect the traffic attacks, where 0 and 1 represent benign and attacks, respectively. We utilized widely used tree-based machine learning models: DT, RF, XGBoost, and CatBoost. The motivations for choosing multiple classification algorithms in developing our tree-based IDS include but are not limited to the following: tree-based models are more computationally efficient than other machine learning methods such as KNN, ANN, or SVM. Also, they are more adaptable in real-time scenarios that require a model update to incorporate the most recent knowledge [45].

Our proposed model employs the following ML techniques:

- **Decision Tree**
  The Decision Tree classifier operates using the divide-and-conquer strategy. This classifier is categorized as non-parametric supervised learning [46]. By employing Decision Tree, it can distinguish between pure and impure subsets of data [47]. The procedure is terminated after the pure subsets have been acquired, or it is repeated.
- **Random Forest**
  The Random Forest classifier was defined by Breiman in 2001. Fernández-Delgado et al. in [48] emphasized that Random Forest is a remarkably efficient classification method for several practical issues. It is comprised of a forest of Decision Trees. This classifier builds Decision Trees by randomly selecting data and making predictions for each tree.
- **XGBoost**
  XGBoost is a newer tree classifier that can scale to large-scale data [49]. This technique has gained wide acceptance in various fields, such as cyber security, due to its exceptional effectiveness and high performance [50]. XGBoost enhances accuracy by combining multiple Decision Trees and reducing the processing time [47].
- **CatBoost**
  The CatBoost algorithm is a potent machine learning technique that produces exceptional results in various applications. Even though CatBoost is primarily intended to manage category features, it can also handle continuous or numerical attributes. The CatBoost model is a unique feature integrated into the gradient-boosting Decision Tree technique [32].
  Overall, Decision Tree classifiers offer a straightforward and comprehensible approach to intrusion detection, allowing analysts to comprehend the decision-making process and identify important factors that affect classification. They provide valuable insights for detecting and dealing with security issues in networks.

**Figure 7:**
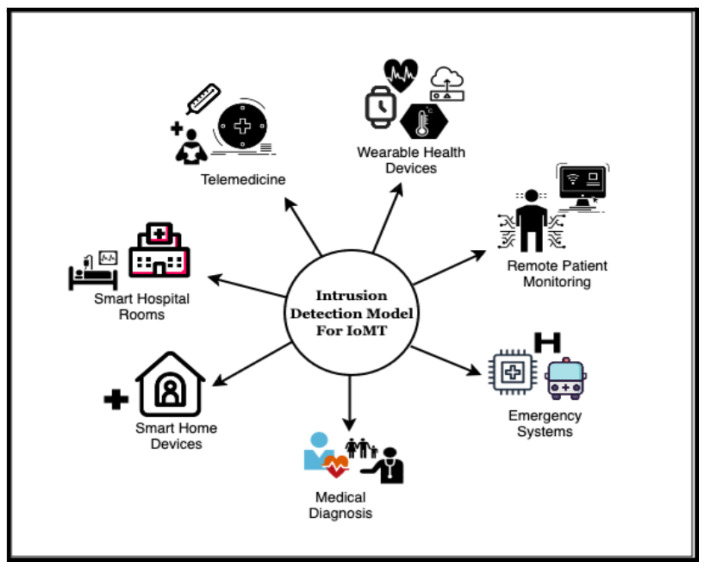
Potential applications of the proposed intrusion detection model in IoMT.

The proposed Intrusion Detection Model utilizes tree-based machine learning classifiers together with feature selection strategies to improve the security of IoMT networks. This approach is applicable in many IoMT environments, where ensuring the accuracy and security of data, as well identifying potential threats, is critical. The model has the ability to be used in various important sectors such as healthcare monitoring, smart home health devices, wearable devices, telemedicine, and emergency response systems. Devices are used in healthcare and related fields, so it is important to think about how our Intrusion Detection Model can be used in these real-life scenarios. This model can be useful in many important areas, including but not limited to maintaining patients in hospitals, making sure that smart health devices used in homes are safe, keeping data safe from wearable health devices. In order to demonstrate the flexibility and real-world importance of the suggested model, Figure 7 presents a graphic representation of potential applications of this model. This graphic illustrates the integration of the model into several IoMT contexts, guaranteeing strong protection against security threats.

## 5. Environment Tools

Python was used to build this method, and the development environment was a Jupyter Notebook made available through Anaconda. Python was chosen for this project because of its wide range of assessment measures, stability, scalability, and efficacy. These factors made Python an excellent choice. The machine learning libraries used in this study include Scikit-learn (version 0.24.2) for the implementation of Decision Trees and Random Forests, XGBoost (version 1.4.2), and CatBoost (version 0.26). To aid in experimentation and analysis, the CICIDS2017 dataset was taken from [37].

## 6. Evaluation Metrics and Results

This section shows the evaluation metrics used to evaluate the performance of the proposed models and the experiment results.

### 6.1. Evaluation Metrics

We employed different evaluation metrics of precision, accuracy, F1-score, recall, receiver operator characteristic (ROC), true positive rate, and false positive rate to assess the effectiveness of the suggested IDS as defined below based on the parameters of false positives (*TP*), true positives (*TP*), false negatives (*FN*), and true negatives (*TN*).

- **Accuracy:** This metric measures the model’s capacity to accurately classify benign instances as displayed in Equation (3):

(3) $$ACC=\frac{TP+TN}{TP+\;TN+\;FP+\;FN}$$

- **Recall:** Also known as the detection rate or sensitivity, this metric measures the model’s ability to recognize attacks as displayed in Equation (4):

(4) $$Recall=\frac{TP}{TP+\;FN}$$

- **Precision:** This metric refers to the model’s capacity to produce accurate predictions, specifically, the number of correctly detected positive predictions (attacks), as displayed in Equation (5):

(5) $$Precision=\frac{TP}{TP+\;FP}$$

- **F1-measure:** This metric effectively addresses the trade-off between recall and precision by balancing them over all instances. This is demonstrated in Equation (6):

(6) $$F1-Measure=\frac{2\times Recall\times Precision}{Recall+\;Precision}$$

- **False positive rate (FPR):** This metric measures the percentage of assault cases classified as normal by the model. This is demonstrated in Equation (7):

(7) $$FPR=\frac{FP}{FP+\;TN}$$

- **True positive rate (TPR):** This metric represents the probability that a true positive will be correctly identified as positive. This is demonstrated in Equation (8):

(8) $$TPR=\frac{TP}{TP+\;FN}$$

- **Receiver operating characteristics area under the curve (ROC AUC)**: This metric works as an indicator of the efficiency of a test across different threshold configurations. A higher level indicates a test that is more useful, with values ranging from 0.0% to 100%. A high ROC value indicates a highly successful classification model. In addition, the evaluation of a model includes the consideration of specificity (TPR) and sensitivity (also known as the true negative rate or TNR), where sensitivity is comparable to recall. This is demonstrated in Equation (9):

(9) $$Specificity=\frac{TN}{FP+\;TN}$$

### 6.2. Experiment Results

The aim of the proposed system is to build a highly efficient IDS framework through the utilization of finely tuned supervised Machine Learning (ML) models. Each model was assessed based on accuracy, precision, recall, and F1-score. As shown in Table 2, all models have good performance. The RF model had a significantly higher accuracy than other classifiers, which was the most accurate at 99.89%, followed by DT and XGBoost models, which had an accuracy of 99.87%. The least accurate model was CatBoost, at 98.79%.

Once the performance of the suggested model is assessed, it is important to validate the models and avoid overfitting. To do this, we employed k-fold cross-validation to validate the models. Different evaluation matrices of the proposed models after the k = 10 cross-validation process are listed in Table 3. It is evident from the table that all models achieve exceptional levels of accuracy, with 15 features chosen using the proposed (MI-XGBoost) feature selection method. According to the accuracy results, CatBoost and XGBoost demonstrate superior performance, achieving average scores of 98.79% and 98.63%, respectively. The least performing model is DT, with 98.12% accuracy. Besides that, the CatBoost model achieves the lowest false alarm rate compared to the other classifiers, with 0.007 FAR.

**Figure 8:**
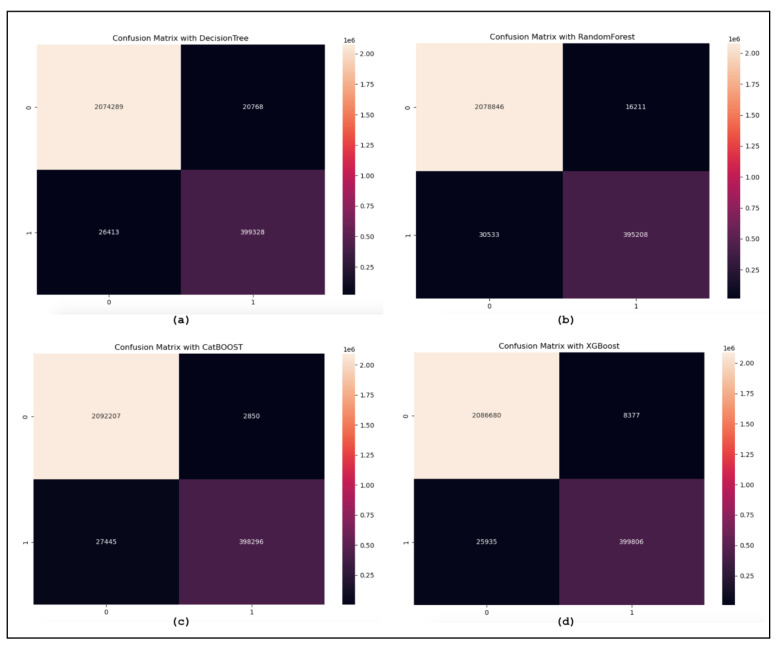
(**a**) The confusion matrix of Decision Tree. (**b**) The confusion matrix of Random Forest. (**c**) The confusion matrix of XGBoost. (**d**) The confusion matrix of CatBoost.

**Figure 9:**
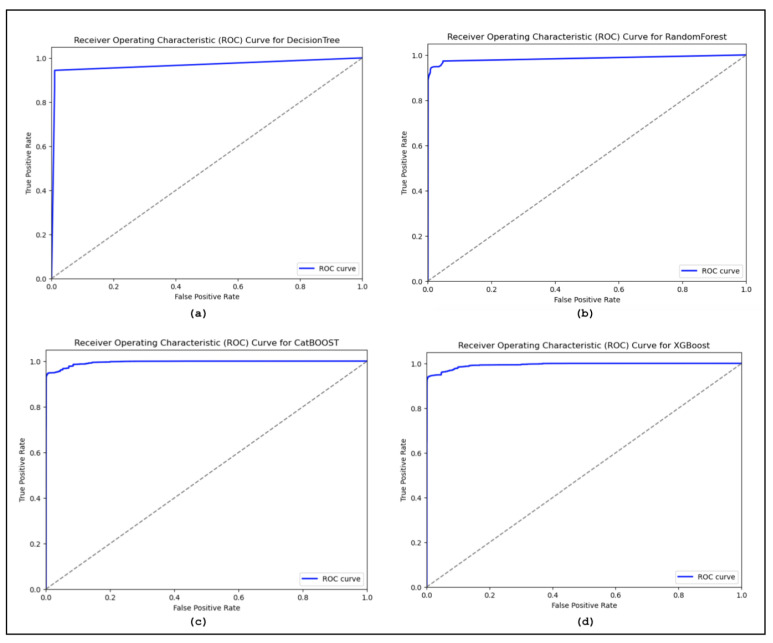
Average ROC curve for (**a**) Decision Tree, (**b**) Random Forest, (**c**) XGBoost, and (**d**) CatBoost.

**Figure 10:**
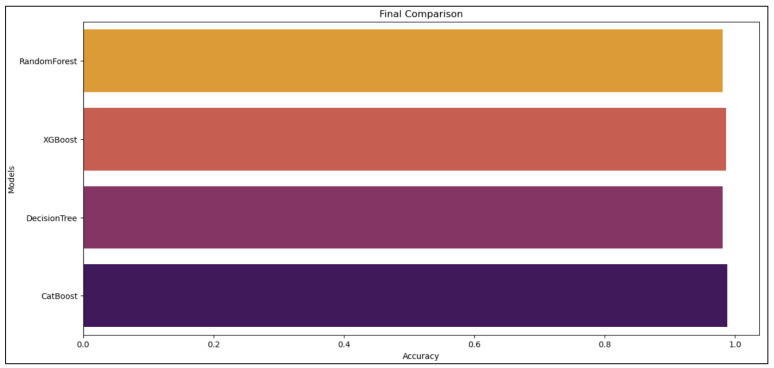
The average of the scores for the accuracy of the proposed models.

The confusion matrices are utilized to assess the proposed model and categorize the traffic as benign or malicious. Figure 8 displays the confusion matrices for the DT, RF, XGBoost, and CatBoost models. Additionally, the confusion matrices reveal the models’ bias for benign samples. More specifically, the models are excessively adapted to the malicious samples to different degrees. The ROC curve is used for sensitivity analysis on classification models. Figure 9 shows the ROC curves illustrating the results of our suggested model using MI-XGBoost filter-based features. Figure 10 shows the average accuracy of our tree-based ML classifiers.

## 7. A Comparison of the Proposed Model with Other State-of-the-Art Methods

In this section, to present the contribution of the proposed system, we compared our tree-based ML model with current state-of-the-art approaches. Table 4 demonstrates that the proposed model overcomes existing classifiers that have utilized the same dataset. As shown in the table, our work is the only work that considers IoMT compared to the other works. Also, our work address using a balanced dataset along with the work in [51] with higher accuracy. Deep comparison of the proposed model with other state-of-the-art methods is shown bellow.

The authors in [26] used a similar dataset with 30 features. To improve the performance of their model, they need to eliminate more features through attribute reduction; in contrast, our model effectively reduced the feature set to 15 features using a feature selection process. Utilizing an eliminated feature set offers advantages in decreasing the model’s complexity level and enhancing the accuracy of detecting the test data. Thus, this reduction leads to faster processing times, which are crucial for real-time applications in IoMT environments.

The study [52] produced a high accuracy rate with 99.98%; however, this result was obtained using an imbalanced dataset.

If the dataset is imbalanced, the model’s high accuracy may not correctly indicate the model’s capacity for generalization to new, unknown data. Accuracy alone does not measure the model’s performance on minority classes, potentially leading to a misleading perception of its usefulness. Nevertheless, our model, which was evaluated using a balanced dataset, achieved high accuracy with 98.79%. This accuracy, though slightly lower, is more indicative of the model’s real-world performance.

Additionally, our suggested model has higher accuracy than the works in [51,53], which obtained an accuracy of 98.12% and 97.23%, respectively.

The higher accuracy of our model (98.79%) is attributed to the combined use of Mutual Information and XGBoost for feature selection and our tree-based ML model for classification, which results in enhanced model detection accuracy.

## 8. Conclusions

In this paper, we proposed a machine learning model for intrusion detection that combines tree-based machine learning with filter-based feature selection techniques. Specifically, the feature selection methods used are Mutual Information and XGBoost Feature Selections (MI-XGBoost). To further reduce the selected features, we utilized the model method to identify 15 features by intersecting the subsets obtained from the top 30 ranked features of (MI-XGBoost) on the CICIDS2017 dataset. Then, we built and assessed the efficacy of four machine learning models, Decision Tree, Random Forest, XGBoost, and CatBoost to distinguish between benign and malicious traffic. The suggested model effectively minimizes the dimension and reduces the number of features to enhance the speed of the classification process while maintaining accuracy. The model achieves remarkable binary classification performance with 98.79% accuracy and a 0.007 FAR.

Our proposed method is built to handle real-time intrusion detection, given the crucial nature of IoMT. Through the utilization of effective algorithms, efficient tree-based algorithms are used to classify the traffic in the network.

To enhance real-time performance, we implemented an optimized feature selection process and the refined feature selection method, thus reducing the number of features, reducing the dimensionality of the data, and quickly processing vast amounts of data, guaranteeing timely detection. Ensuring the security and integrity of IoMT networks requires this capability.

Our model outperformed other state-of-the-art works by reducing the number of features to 15 while achieving a high accuracy of 98.79%. Although one model achieved higher accuracy, they used imbalanced datasets, making our model which used balanced data more reliable for intrusion detection in IoMT environments. In addition, the proposed tree-based classifiers enhanced stability and the handling of huge data. The results obtained from our model demonstrate that the integration of tree-based classifiers along with filter-based feature selection techniques further contributes to the model’s ability to handle and detect intrusions in the IoMT.

In future work, while this work focuses on the binary classification for intrusion detection, we will consider designing a multi-classification approach to identify and categorize various types of attacks. Furthermore, we intend to apply our suggested feature selection strategy to other machine learning models. In addition, while our proposed model has demonstrated strong performance in simulations using the CICIDS2017 dataset, we recognize the importance of validating our approach under real-world conditions. Future work will focus on conducting empirical testing within practical IoMT environments to assess the model’s robustness, scalability, and real-time performance. This will involve deploying the model in a live network setting, where factors such as network latency, computational resources, and dynamic data streams can be thoroughly evaluated.

## Figures and Tables

**Table 1:** The day activity and the traffic type for each file.

Day Activity	Traffic Type
Monday	Benign
Tuesday	Benign, FTP-Patator, SSH-Patator
Wednesday	Benign, DoS GoldenEye, DoS Hulk, Dos Slowhttptest, Dos Slowlories, Heartbleed
Thursday (Morning)	Benign, Web Attack XSS, Web Attack Brute Force, Web Attack Sql Injection
Thursday (Afternoon)	Benign, Infilration
Friday (Morning)	Benign, Botnet.
Friday (Afternoon)	Benign, PortScan
Friday (Afternoon)	Benign, DDos

**Table 2:** The valuation results of the proposed model’s detection performance using MI-XGBoost features.

Model	Accuracy	Precision	Recall	F1 Score
DT	0.9987	0.9948	0.9980	0.9964
RF	0.9989	0.9954	0.9981	0.9967
XGBoost	0.9987	0.9957	0.9970	0.9963
CatBoost	0.9969	0.9829	0.9990	0.9909

**Table 3:** The average comparison of different evaluation matrices of the proposed models after ten-fold cross-validation.

Model	Accuracy %	ROC	DR %	FAR
DT	98.12	0.9505	98.74	0.049
RF	98.14	0.960	98.55	0.039
XGBoost	98.63	0.992	98.77	0.0205
CatBoost	98.79	0.9794	98.70	0.007

**Table 4:** Our proposed model compared to other state-of-the-art methods.

Study	Year	Dataset	Balanced	FS Methods	Num of FS	ML-Classifier	Accuracy	IoMT	Cross-Validation
[26]	2022	CICIDS2017	×	(CFS–FPA)	30	SVM, RF, NB and KNN	99.7	×	✓
[52]	2022	CICIDS2017 NSL-KDD	×	RFE (best FST)	8	DT, NB, and KNN	99.98	×	✓
[51]	2024	CICIDS2017	✓	ANOVA	10	SVC, DT, RF, GNB, AdaBoost, XGBoost and LR	97.23	×	×
[53]	2024	CICIDS2017	-	-	-	RF and DT	98.12	×	✓
Our work	2024	CICIDS2017	✓	MI-XGboost	15	DT, RF, XGboost, and Catboost	98.79	✓	✓

Note: - indicate not mentioned in the study.

## Data Availability

Data are available upon request.
